# Predicting functional performance via classification of lower extremity strength in older adults with exergame-collected data

**DOI:** 10.1186/s12984-020-00778-z

**Published:** 2020-12-10

**Authors:** Hagen Becker, Augusto Garcia-Agundez, Philipp Niklas Müller, Thomas Tregel, André Miede, Stefan Göbel

**Affiliations:** 1 Multimedia Communications Lab, Technische Universitaet Darmstadt, Rundeturmstr. 10, 64283 Darmstadt, Germany; 2 HTW Saar, Saarbruecken University of Applied Sciences, Saarbrücken, Germany

**Keywords:** Wii Balance Board, Physical independence, Balance, Exergames, Serious games

## Abstract

**Objective:**

The goal of this article is to present and to evaluate a sensor-based functional performance monitoring system. The system consists of an array of Wii Balance Boards (WBB) and an exergame that estimates whether the player can maintain physical independence, comparing the results with the 30 s Chair-Stand Test (30CST).

**Methods:**

Sixteen participants recruited at a nursing home performed the 30CST and then played the exergame described here as often as desired during a period of 2 weeks. For each session, features related to walking and standing on the WBBs while playing the exergame were collected. Different classifier algorithms were used to predict the result of the 30CST on a binary basis as able or unable to maintain physical independence.

**Results:**

By using a Logistic Model Tree, we achieved a maximum accuracy of 91% when estimating whether player’s 30CST scores were over or under a threshold of 12 points, our findings suggest that predicting age- and sex-adjusted cutoff scores is feasible.

**Conclusion:**

An array of WBBs seems to be a viable solution to estimate lower extremity strength and thereby functional performance in a non-invasive and continuous manner. This study provides proof of concept supporting the use of exergames to identify and monitor elderly subjects at risk of losing physical independence.

## Introduction

Falls are an important cause of mortality and early placement in nursing homes in older adults. The main causes of falls are accidental and environment-related (31%), or caused by gait imbalance (17%). Approximately 30 to 60% of older adults fall each year. Out of these falls, 10 to 20% result in injury, hospitalization, or death. Risk assessment and exercise are among the most relevant factors to prevent these falls [1]. The role of sensor-based solutions in regards to falling risk has traditionally been focused on detecting said falls. Both wearable and smartphone-based solutions for fall detection are readily available for this purpose [2]. Although this approach is useful, detecting elderly who are at risk of losing physical independence, and thus may fall in the near future, would provide an additional method to prevent falls before they occur.

Exergames are active video games that incorporate physical movements, aiming to combine physical exercise with the fun associated with gaming. The main advantage of using exergames is that they increase motivation and thus adherence to training [3]. These exergames can be designed to require players to perform physical movements similar to those of fall risk prevention exercises. At the same time, and in the background, data of clinical relevance can be collected from the sensors used to control the exergame, [4]. Furthermore, it is also possible to adapt the exergame to the specific needs of the user in real-time and without external intervention, based on how players perform in the game [5]. This holds promise for using exergames as rehabilitation tools able to provide continuous physical improvement [6].

The potential of the Wii Balance Board (WBB) to estimate whether the player can maintain physical independence has already been identified [7]. However, the relationship between WBB data and the estimation of clinically meaningful physical independence metrics is unclear. In this sense, Mertes et al. discussed that WBB data contain information that allows discrimination between elderly who previously fell and others who did not [8]. Their study achieved an accuracy of 76.6% when classifying fallers and non-fallers among 12 participants. Early evidence also shows that the WBB could be used to train balance in the elderly [9], and that there are statistically significant differences in the way elderly at falling risk interact with the WBB as compared to individuals with no falling risk. These differences correlate with clinical fall risk tests, further supporting our hypothesis that a direct relation between WBB data and clinical metrics for physical independence can be established. Yamada et al. [10] found statistically significant differences and moderate correlations (r = 0.69) in a study with 45 participants.

A limitation of the WBB is that, due to its small surface, it can only be used to estimate balance while standing, but not in movement. In a previous article, we presented PDDanceCity, a city map exergame that provides dual-tasked cognitive and physical rehabilitation [11]. The game is controlled with an array of six WBBs, which we call Extended Balance Board (EBB) [12]. Thanks to its extended surface, EBB data can be used to estimate the balance of the player both while standing and walking. We believe the data extracted from the EBB could be used to estimate the balance and gait skills of the player in the background, without the need to actively perform any specific test, or for any caregiver to be present.

To do so, this study aims to analyze the possibility of training a classifier to predict the clinical functional performance of a player based on EBB data collected in the background while playing PDDanceCity. This can be achieved by attempting to predict the score of a standardized test that can be used to assess the capability of maintaining physical independence. There are several such tests to measure lower extremity strength, for example, the 30-s Chair-Stand test (30CST) [13], which is part of the Fullerton Fitness Test Battery, and is fairly easy to administer. The Fullerton Fitness Test Battery is commonly employed in older adults in community settings. It can measure physical patterns of physical decline in advanced ages. Evidence suggests it could also be used as a screening test to estimate the balance impairment in older adults [14, 15]. The 30CST classifies participants as subjects able or unable to maintain physical independence, depending on whether their test score is above or below an age- and sex-adjusted cutoff. We hypothesize this binary prediction could be achieved with a classifier algorithm using data extracted from the EBB.

The goal of this study is to determine the potential of classifying EBB-extracted data to perform a binary prediction, that is, whether the player is able or unable to maintain physical independence. This prediction could be used to detect when individuals are at increased risk of losing physical independence and could be more prone to fall in the near future. We aim to validate this estimation basing the result on a prediction of the 30CST score. Data is collected while users are playing PDDanceCity to provide a very simple background screening process determining whether the player may be unable to maintain physical independence.

## Methods

**Fig. 1:**
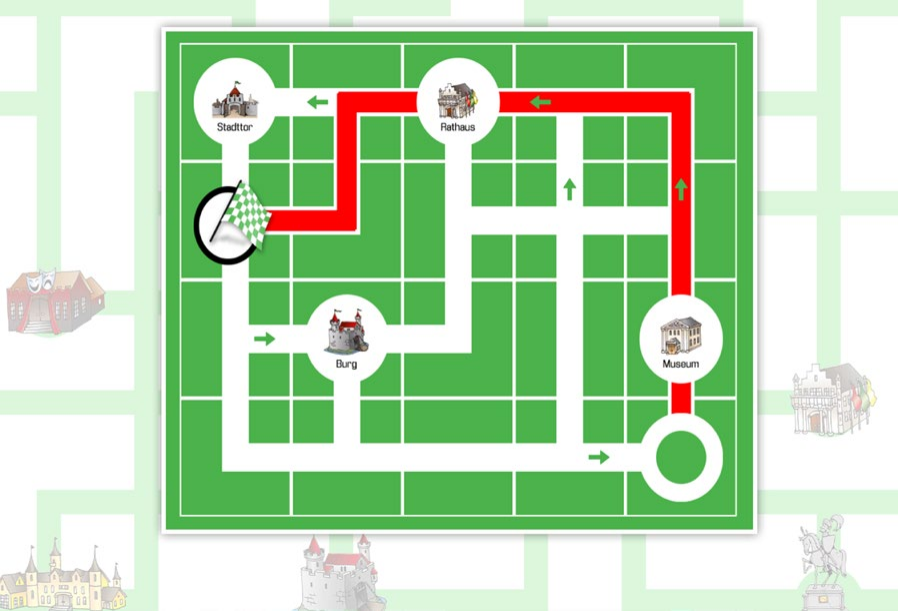
PDDanceCity exergame

PDDanceCity [11] is a labyrinth navigation exergame designed for dual-tasking rehabilitation. The goal of the game is to navigate the labyrinth, representing a city map, to reach a goal, where only two-dimensional movements are possible (up, down, right, and left). As an additional requirement, players are encouraged to reach the target with the least possible number of steps. Besides, they may be required to visit a given number of points of interest (for example a museum, monument, or café) which may, or may not, be directly on the shortest path (Fig. 1). The game offers dual-tasking rehabilitation, training visuospatial function, memory, balance, and physical coordination.

**Fig. 2:**
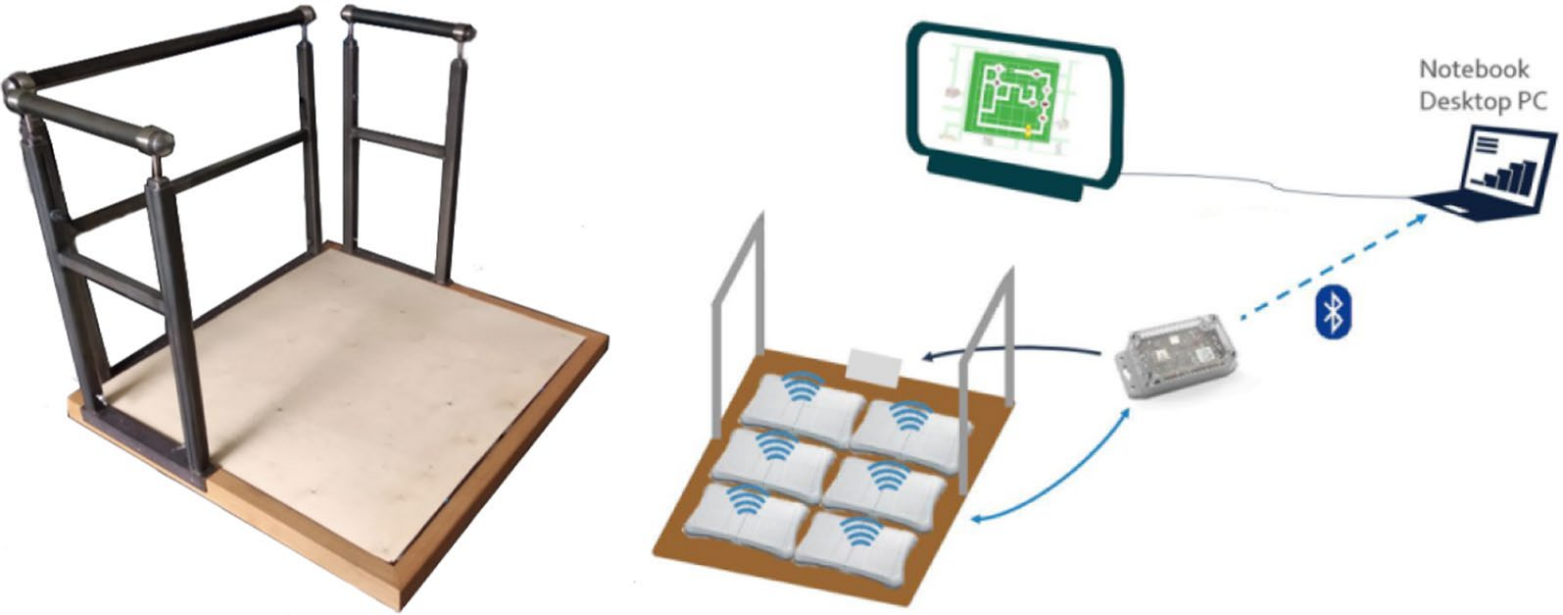
PDDanceCity scenario setup

PDDanceCity is controlled with a system consisting of an array of six WBBs, called EBB [12] (Fig. 2). A controller receives all data from the WBBs and forwards it via a USB connection to a PC. Information sent through the USB interface contains the board identifier (ID), based on its MAC address, as well as the current value of each of its weighing sensors (four per WBB, for a total of 24). The refresh rate per board is 20 Hz.

**Fig. 3:**
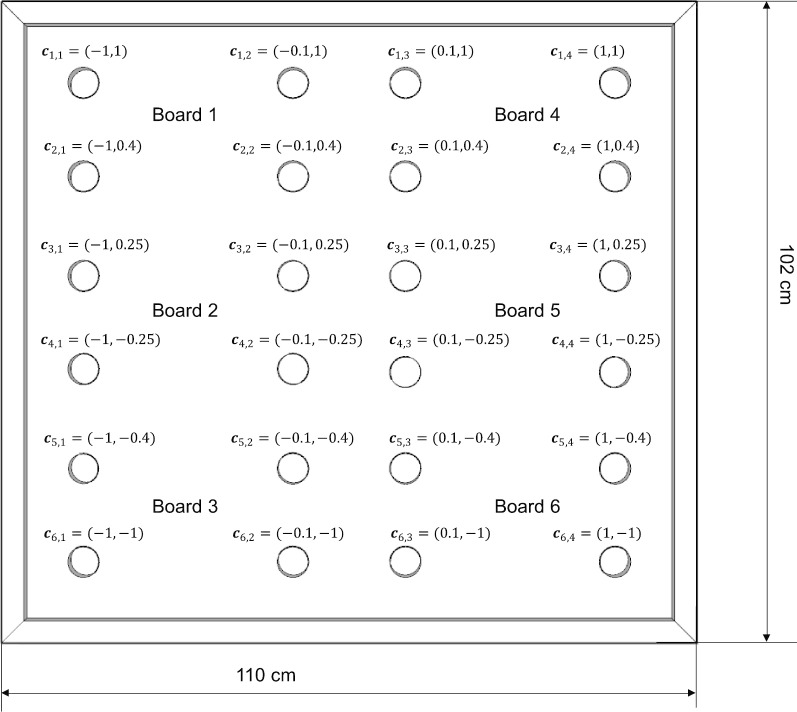
EBB coordinate system

To use EBB data to control PDDanceCity, the center of mass ${\varvec{c}}{\varvec{o}}{\varvec{m}}(t)$ is calculated as follows. We define $\mathbf{S}$ as the 6 × 4 matrix of sensor values (six WBB boards and four sensors per board), and ${s}_{\mathrm{i},\mathrm{j}}\left(t\right)$ as the value of sensor $\left(i,j\right)$ of $\mathbf{S}$ in instant $t$. We define $\mathbf{C}$ as the matrix of $(x,y)$ coordinate vectors ${{\varvec{c}}}_{\mathrm{i},\mathrm{j}}$ assigned to each sensor (Fig. 3), based on its position. We also define $w\left(t\right)$ as the last total weight value calculated by all boards, that is, the weight of the player. ${\varvec{c}}{\varvec{o}}{\varvec{m}}(t)$ is calculated as the weight-normalized bidimensional projection of sensor values as:

$${\varvec{c}}{\varvec{o}}{\varvec{m}}(t)={(com}_{x}(t),{com}_{y}(t))=\frac{1}{w(t)} {\sum\limits_{i=1}^{6}}{\sum\limits_{j=1}^{4}}{(s}_{\mathrm{i},\mathrm{j}}\left(t\right){{\varvec{c}}}_{\mathrm{i},\mathrm{j}})$$

**Fig. 4:**
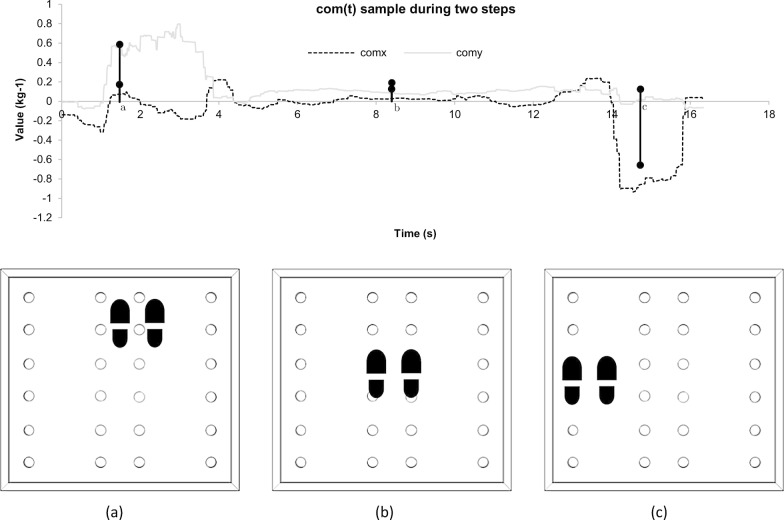
Calculation of the center of mass (top) and position of the feet (bottom) during a step forward (**a**), while standing on the center (**b**) and during a step leftwards (**c**)

This results in a set of two minus-one to one values $$({com}_{x},{com}_{y})$$ which can be used to determine intentionality. To achieve this, we define a directional intention based on two conditions: the main directional component must be equal to or greater than 0.5 in magnitude, and the other component must be equal to or lesser than 0.1 in magnitude. As an example, $$(0.1$$,$$0.9)$$ represents an upwards step, and $$(-0.8$$,$$0.05)$$ would represent a leftwards movement. Between each step, the player is always required to return to the center (both values lower than or equal to 0.1 in magnitude). Figure 4 represents two examples of this directional intention. We also define the instability factor $$if(t)$$ as an approximation of the first-order differential of $${\varvec{c}}{\varvec{o}}{\varvec{m}}(t)$$. This parameter is a measure of how a player shifts their weight on the EBB. A very fast weight shifting, causing a high value of $$if(t)$$, would be an indicator of potential lack of balance (or loss thereof) among older adults who are not expected to move quickly. This is calculated as:

$$if(t)= \sqrt{\frac{1}{2}{({com}_{x}(t)-{com}_{x}(t-1))}^{2}+\frac{1}{2} {({com}_{y}(t)-{com}_{y}(t-1))}^{2}}$$

where $(t-1)$ represents the value prior to the most recent one $t$. In this manner, when $if(t)$ surpasses a certain threshold, a potential loss of balance might have occurred. For every level played, PDDanceCity stores a.xml file that includes the player’s profile information, information about the level, steps taken, and all values of ${\varvec{c}}{\varvec{o}}{\varvec{m}}(t)$ and$if(t)$.

Finally, we extract a series of features based on ${\varvec{c}}{\varvec{o}}{\varvec{m}}(t$) and $if(t)$. These features are mostly related to average values, standard deviations and maxima and minima of ${\varvec{c}}{\varvec{o}}{\varvec{m}}(t$) under different circumstances, as well as the number of times that $if(t)$ overcame different possible thresholds. In addition to these two elements, we also consider features related to the time intervals between steps, and the standard deviation of these intervals. A complete feature list is presented in Table 1. All features are calculated per playthrough, with no windowing. We used the Matlab software to calculate these features [16].

**Table 1:** System features and calculation

Features	Description	Calculation
${Com}_{{Avg}_{Direction}}$	Average ${\varvec{c}}{\varvec{o}}{\varvec{m}}$ value for movements in each direction, where ${n}_{COM,Direction}$ represents the number of steps for each direction. Four two-dimensional features $(x,y)$ per playthrough	$\frac{{\sum }_{t=1}^{{n}_{COM,Direction}}{\varvec{c}}{\varvec{o}}{\varvec{m}}\left(t\right)}{{n}_{COM,Direction}}$ $Direction=Up \leftrightarrow {com}_{y}>0.5, \left|{com}_{x}\right|<0.1$ $Direction=Down \leftrightarrow {com}_{y}<-0.5, \left|{com}_{x}\right|<0.1$ $Direction=Right \leftrightarrow {com}_{x}>0.5, \left|{com}_{y}\right|<0.1$ $Direction=Left \leftrightarrow {com}_{x}<-0.5, \left|{com}_{y}\right|<0.1$
${Com}_{{Std}_{Direction}}$	Standard deviation of ${\varvec{c}}{\varvec{o}}{\varvec{m}}$**,** for each direction, as above. Eight features per playthrough	$\sqrt{\frac{{\sum }_{t=1}^{{n}_{COM,Direction}}{\left({com}_{i}\left(t\right)-{{Com}_{Avg}}_{Direction,i}\right)}^{2}}{{n}_{COM,j}-1}},$ $i=x,y, Direction=Up,Down,Left, Right$
${Balance}_{Up}, {Balance}_{Down}$	Average value of ${com}_{y}$ for all values where ${com}_{y}>0$ (up) or ${com}_{y}<0$ (down), where ${n}_{COM}$ is the number of ${\varvec{c}}{\varvec{o}}{\varvec{m}}$ samples**.** Two features per playthrough	$\frac{{\sum }_{t=1}^{{n}_{COM}}{com}_{y}(t)}{{n}_{COM}}:{com}_{y}>0$, $\frac{{\sum }_{t=1}^{{n}_{COM}}{com}_{y}(t)}{{n}_{COM}}:{com}_{y}<0$
${Balance}_{Right}, {Balance}_{Left}$	Average value of ${com}_{x}$ for all values where ${com}_{x}>0$ (right) or ${com}_{x}<0$ (left). Two features per playthrough	$\frac{{\sum }_{t=1}^{{n}_{COM}}{com}_{x}(t)}{{n}_{COM}}:{com}_{x}>0$, $\frac{{\sum }_{t=1}^{{n}_{COM}}{com}_{x}(t)}{{n}_{COM}}:{com}_{x}<0$
${Avg}_{x}, {Avg}_{y}$	Average value of ${com}_{x}$ and ${com}_{y}$. Two features $(x,y)$ per playthrough	$\frac{{\sum }_{t=1}^{{n}_{COM}}{com}_{x}(t)}{{n}_{COM}}$, $\frac{{\sum }_{t=1}^{{n}_{COM}}{com}_{y}(t)}{{n}_{COM}}$
${Max}_{x}, {Max}_{y}$, ${Min}_{x}, {Min}_{y}$	Maximum and minimum value of ${com}_{x}$ and ${com}_{y}$. Two features $(x,y)$ per playthrough	$Max ({com}_{x}\left(t\right),\forall t)$, $Max ({com}_{y}\left(t\right),\forall t)$, $Min ({com}_{x}\left(t\right),\forall t)$,$Max ({com}_{y}\left(t\right),\forall t)$
${Std}_{x}, {Std}_{y}$	Standard deviation of ${com}_{x}$ and ${com}_{y}$. Two features $(x,y)$ per playthrough	$\sqrt{\frac{{\sum }_{t=1}^{{n}_{COM}}{\left({com}_{i}\left(t\right)-{Avg}_{i}\right)}^{2}}{{n}_{COM}-1}}, i=x,y$
${If}_{Avg}$, ${If}_{Max}$	Average $\mathrm{i}f\left(t\right)$ value and maximum for the whole playthrough. Two features per playthrough	$\frac{{\sum }_{t=1}^{{n}_{COM}}if(t)}{{n}_{COM}}$, $Max (\mathrm{i}f\left(t\right),\forall t)$
${If}_{Threshold,i}$	Number of times $\mathrm{i}f\left(t\right)>i, i=\left[\mathrm{0.5,1},\mathrm{1.5,2}\right].$ Normalized by the total number of samples. Four features per playthrough	$\frac{N (if(\mathrm{t})>i)}{{n}_{COM}}, i=\mathrm{0.5,1},\mathrm{1.5,2}$
${If}_{{Sum}_{Avg}}$, ${If}_{{Sum}_{Max}}$	Average value and maximum of the sum of the last 25 values of $\mathrm{i}f\left(t\right)$ for the whole playthrough. Two features per playthrough	$\frac{{\sum }_{t=1}^{{n}_{COM}}{if}_{Sum}(t)}{{n}_{COM}}, {if}_{Sum}\left(t\right)={\sum }_{i=t-24}^{t}if(\mathrm{t})$, $Max ({if}_{Sum}(t),\forall t)$
${If}_{{Sum}_{Overx}}$	Number of times ${If}_{Sum}\left(t\right)>i,i=\left[\mathrm{0.5,1},\mathrm{1.5,2}\right].$ Normalized by total playthrough time. Four features per playthrough	$\frac{N ({if}_{Sum}(t)>i)}{{n}_{COM}}, i=\mathrm{0.5,1},\mathrm{1.5,2}$
${Step}_{Avg}$	Average time between steps, excluding the first step, defining ${Step}_{Time}(i)$ as the time in seconds in which step $i$ occurred, and ${n}_{Steps}$ as the total number of steps in the playthrough. One feature per playthrough	$\frac{{\sum }_{i=2}^{{n}_{Steps}}{Step}_{Time}\left(i\right)-{Step}_{Time}(i-1)}{{n}_{Steps}}$
${Step}_{Std}$	Standard deviation of time between steps, excluding the first step. One feature per playthrough	$\sqrt{\frac{{\sum }_{i=2}^{{n}_{Steps}}{\left({Step}_{Time}\left(i\right)-{Step}_{Time}(i-1)-{Step}_{Avg}\right)}^{2}}{{n}_{Steps}-1}}$

To evaluate our system, we recruited 16 participants (median age 73, 6 males) at a nursing home in Darmstadt, Germany. A computer was installed in a common room, connected to a television and the EBB (Fig. 2). Participants were invited to play PDDanceCity as often as they desired for a period of 2 weeks. During the first session, nominal data (age and sex) was collected, and the 30CST was administered. The resulting 30CST scores ranged between 0 and 17, with a median of 13. All sessions took place under observation of one of the authors, to ensure that no falls occurred. Otherwise, the game sessions were unsupervised. We obtained the approval of the ethics committee of the Technical University of Darmstadt for this evaluation.

In total, these 16 participants played 87 levels of PDDanceCity during this period. The median number of levels per participant was 5. Each level of PDDanceCity takes approximately 2 to 3 min to complete, resulting in an approximated gameplay time of 10 to 15 min per participant. For each level, a single training instance was obtained. The data of 6 of these levels had to be discarded due to data failure, leaving 81 training instances for classification. Due to the reduced number of participants, and to minimize the risk of overfitting based on age and sex, we attempted to classify if the player’s predicted 30CST score was above or below a cutoff score of 12 points, without using these nominal data (age and sex) as features. We refer to players classified above this cutoff as fit, and those under the cutoff as not fit. This score was chosen to even out both groups, as eight participants had a 30CST score of 11 or lower. We also explore the possibility of predicting the adjusted cutoffs, which we discuss at the end of “Results” section. All classification tasks were performed using Weka [17].

## Results

**Fig. 5:**
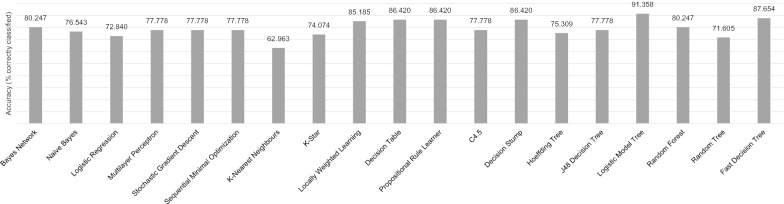
Classification accuracies

The best classification results are presented in Table 2. This decision tree used the average time between steps exclusively, with a score of 6.17 or lower, indicating a participant able to maintain physical independence. A comparison of different classification algorithms is presented in Fig. 5. In all cases, we performed our classification using ten-fold cross-validation. Results of a feature selection analysis (information gain attribute evaluation) are included in Table 3. No features were excluded for classification.

**Table 2:** Best classification results using a Logistic Model Tree

Algorithm: Logistic Model Tree, accuracy 91.358%	Correctly classified	Incorrectly classified	TP rate	FP rate	Precision	Recall	F	MCC	ROC area	PRC area
Not fit	29 (TP)	5 (FN)	0.853	0.043	0.935	0.853	0.892	0.823	0.940	0.946
Fit	45 (TN)	2 (FP)	0.957	0.147	0.900	0.957	0.928	0.823	0.940	0.930
Weighted average	74	7	0.914	0.103	0.915	0.914	0.913	0.823	0.940	0.936

*TP* true positive, *FP* false positive, *F* F-measure, *MCC* Matthews correlation coefficient, *ROC* receiver-operating characteristic curve, *PRC* precision-recall curve

**Table 3:** Information gain attribute results based on delta entropy information gain

Feature	Information gain (∆ Entropy)
${Step}_{Avg}$	0.486
${If}_{{Sum}_{Over0.5}}$	0.321
${If}_{{Sum}_{Over1}}$	0.241
${If}_{Threshold,0.5}$	0.178
${Step}_{Std}$	0.145
${If}_{Avg}$	0.14
${If}_{{Sum}_{Avg}}$	0.14
${If}_{Max}$	0.138
${Balance}_{Left}$	0.118

As a second potential scenario of analysis, we also aimed to predict the age- and sex-adjusted 30CST cutoff scores. The resulting accuracy was very high (99%) but, as discussed in the previous section, we suspect that to be due to overfitting to age and sex because of our limited sample size, as the classifier did achieve 100% accuracy using exclusively age and sex as features. If we remove these two features in this scenario, we achieve a classification accuracy of 86% predicting the age- and sex-adjusted 30CST outcome. For this reason, we believe that provided a large (and diverse) enough sample size of participants of a wide array of ages and different degrees of fitness, it should be possible to predict the age- and sex-adjusted 30CST binary result using the methods presented in this publication.

We complemented this classification with the analysis of the effect sizes of each feature between the fit and not fit groups, measured on the basis of Hedges’ g, due to the low sample size and the disparity in standard deviations. We also evaluated statistical significance using a Welch t-test. These effect sizes are presented in Table 4. Features related to the instability factor and the mean and standard deviation of the time between steps, seem to contain the information most related to the 30CST. Following Cohen’s rule of thumb (0.2 is a small, 0.5 a medium and 0.8 a large effect size), the effect sizes of these features are large, with the differences between the fit and not fit groups being in most cases very significant (p < 0.001) or at least significant (p < 0.05).

**Table 4:** Effect size and statistical significance of features

Feature	Not fit-fit effect size (g)	Not fit-fit significance (p)	Feature	Not fit-fit effect size (g)	Not fit-fit significance (p)
${Com}_{{Avg}_{Up},x}$	0.3168	0.1998	${Avg}_{y}$	− 0.0056	0.9817
${Com}_{{Avg}_{Up},y}$	− 0.4678	0.0595	${Max}_{x}$	− 0.3507	0.1528
${Com}_{{Avg}_{Down,x}}$	0.2790	0.2692	${{\varvec{M}}{\varvec{a}}{\varvec{x}}}_{{\varvec{y}}}$	− **0.5279**	**0.0310**
${Com}_{{Avg}_{Down,y}}$	− 0.1171	0.6238	${Min}_{x}$	0.3565	0.1472
${Com}_{{Avg}_{Right},x}$	− 0.2328	0.3404	${Min}_{y}$	− 0.1036	0.6630
${Com}_{{Avg}_{Right},y}$	− 0.4073	0.1028	${{\varvec{S}}{\varvec{t}}{\varvec{d}}}_{{\varvec{x}}}$	− **0.6665**	**0.0068**
${Com}_{{Avg}_{Left},x}$	0.2661	0.2756	${Std}_{y}$	− 0.3789	0.1247
${Com}_{{Avg}_{Left},y}$	− 0.3234	0.1863	${{\varvec{I}}{\varvec{f}}}_{{\varvec{A}}{\varvec{v}}{\varvec{g}}}$	− **0.7478**	**0.0035**
${Com}_{{Std}_{Up,x}}$	− 0.0323	0.8966	${{\varvec{I}}{\varvec{f}}}_{{\varvec{M}}{\varvec{a}}{\varvec{x}}}$	− **0.6337**	**0.0119**
${{\varvec{C}}{\varvec{o}}{\varvec{m}}}_{{{\varvec{S}}{\varvec{t}}{\varvec{d}}}_{{\varvec{U}}{\varvec{p}},{\varvec{y}}}}$	− **0.4913**	**0.0461**	${{\varvec{I}}{\varvec{f}}}_{{\varvec{T}}{\varvec{h}}{\varvec{r}}{\varvec{e}}{\varvec{s}}{\varvec{h}}{\varvec{o}}{\varvec{l}}{\varvec{d}},0.5}$	− **0.7452**	**0.0024**
${Com}_{{Std}_{Down,x}}$	0.2234	0.3490	${If}_{Threshold,1}$	− 0.2411	0.2873
${Com}_{{Std}_{Down,y}}$	0.4173	0.0860	${If}_{Threshold,1.5}$	0	0
${Com}_{{Std}_{Right,x}}$	0.0318	0.8977	${If}_{Threshold,2}$	0	0
${Com}_{{Std}_{Right,y}}$	0.0753	0.7621	${{\varvec{I}}{\varvec{f}}}_{{{\varvec{S}}{\varvec{u}}{\varvec{m}}}_{{\varvec{A}}{\varvec{v}}{\varvec{g}}}}$	− **0.7387**	**0.0038**
${Com}_{{Std}_{Left,x}}$	− 0.3164	0.1959	${If}_{{Sum}_{Max}}$	− 0.2107	0.3938
${Com}_{{Std}_{Left,y}}$	− 0.0237	0.9217	${{\varvec{I}}{\varvec{f}}}_{{{\varvec{S}}{\varvec{u}}{\varvec{m}}}_{{\varvec{O}}{\varvec{v}}{\varvec{e}}{\varvec{r}}0.5}}$	− **1.5261**	**< 0.0001**
${Balance}_{Up}$	0.1598	0.5215	${{\varvec{I}}{\varvec{f}}}_{{{\varvec{S}}{\varvec{u}}{\varvec{m}}}_{{\varvec{O}}{\varvec{v}}{\varvec{e}}{\varvec{r}}1}}$	− **0.9196**	**0.0003**
${Balance}_{Down}$	0.1623	0.4988	${If}_{{Sum}_{Over1.5}}$	− 0.2206	0.3477
${Balance}_{Right}$	− 0.3628	0.1429	${If}_{{Sum}_{Over2}}$	− 0.2062	0.3762
${{\varvec{B}}{\varvec{a}}{\varvec{l}}{\varvec{a}}{\varvec{n}}{\varvec{c}}{\varvec{e}}}_{{\varvec{L}}{\varvec{e}}{\varvec{f}}{\varvec{t}}}$	**0.6306**	**0.0091**	${{\varvec{S}}{\varvec{t}}{\varvec{e}}{\varvec{p}}}_{{\varvec{A}}{\varvec{v}}{\varvec{g}}}$	**1.2260**	**< 0.0001**
${Avg}_{x}$	0.1925	0.4267	${{\varvec{S}}{\varvec{t}}{\varvec{e}}{\varvec{p}}}_{{\varvec{S}}{\varvec{t}}{\varvec{d}}}$	**0.8446**	**0.0020**

All values are presented as not fit vs. fit, meaning that a negative effect size indicates the parameter has lower values in the not fit group. According to Cohen’s rule, g > 0.8 indicates a large effect size. Bold emphasis indicates statistical significance (p < 0.05)

## Discussion

Despite the limited number of participants and training instances, we obtained excellent classification results. Generally, decision trees seem to provide the best performance in the proposed classification task.

Although we decided on using the 30CST to minimize the risk of falls while conducting the test, such test is correlated to physical independence, but not the risk of falling. This is a limitation of this study since, in order to evaluate the feasibility of using EBB data to predict the risk of falling directly, an alternative assessment method, such as the Berg Balance Scale (BBS), should be used. A future study with a larger cohort should consider using the BBS instead of the 30CST to further support the hypothesis that EBB data can be used to accurately identify participants at an increased falling risk. In addition, further balance-related data from participants (Physiological Profile Assessment, functional balance, gait speed, or prior falls) should be collected as well.

Our design also presents some technical limitations. At the moment, the WBBs send the data via Bluetooth, which means they have to be manually connected for each play session. They also operate on batteries, and when these are low the data received is not reliable anymore, thus leading to data failure. Additionally, the EBB frame presents a risk depending on how the EBB is placed in its surroundings: if it is not set against a wall behind it, a player may fall when taking a step backward. We aim to address these technical limitations in a future iteration of the EBB by providing direct electrical supply to the WBBs, automating the Bluetooth synchronization process, and building a complete enclosure around the EBB.

## Conclusions

This study provides proof of concept supporting the use of exergames to identify elderly subjects at risk of losing physical independence. Despite the aforementioned limitations, our results suggest that the EBB, as an extension of the WBB, can be used to screen the elderly population for individuals which are likely to lose physical independence in the near future, thus guiding therapeutic and rehabilitation adjustments. Nevertheless, a larger dataset is required to determine the feasibility of predicting if a participant will be above or below their age- and sex-adjusted 30CST cutoff score. This could also open the possibility of predicting the result of similar tests, such as the Berg Balance Scale or the Ten-Meter Walk Test. Once the technical limitations of the EBB are addressed, and considering that participants played without supervision, a home (or, more generally, unsupervised) scenario seems feasible. In the future, we aim to extend our evaluation including features related to game performance, conducting a similar evaluation concerning cognition. This could be done, for example, on the basis of the Mini-Mental State Examination.

## Data Availability

Due to the nature of the data, the feature datasets of the presented classification are available from the corresponding author on reasonable request.
